# Junius H. Wattles, Sr., M.D., D.V.S.

**Published:** 1902-12

**Authors:** 


### NECROLOGY.

### JUNIUS H. WATTLES, SR., M.D., D.V.S.

THE death of Dr. Junius H. Wattles, Sr., of Kansas City, Mo., removes an active figure from the veterinary profession.

Dr. Wattles went to Kansas City several years ago from Kenosha, Wisconsin, and closed his earthly career in the city of his adoption November 8, 1902. His death ensued from ptomaïne poisoning, following the eating of some calves' brains.

He was the founder of the Kansas City Veterinary College, in 1891, and remained at its head as chief instructor until it was purchased by the present officers of the institution. He maintained it as a two-year school during his connection therewith. On relinquishing his interest in the Kansas City Veterinary College he pursued a course in human medicine and added the degree of human medicine to his veterinary title, which was obtained at the Chicago Veterinary College in the year 1887.

Dr. Wattles did not find human medicine so attractive a field as that of comparative science, and returned to the practice of veterinary medicine, and, with little to aid him or encourage the enterprise, again embarked in the development of a second veterinary college in Kansas City, Mo. This institution, known as the Western Veterinary College, he conducted up to the time of his death, which, after maintaining it for several years under the most meagre facilities, he succeeded at the opening of the present college year in housing the institution in a building specially constructed for this purpose.

He was a hard worker and aggressive in all his undertakings, and unrelenting as a foe with those from whom he felt he received any injury.

He leaves a wife who barely escaped death from the same cause, and one son, Junius H. Wattles, Jr., a graduate in veterinary science, who mildly suffered from a similar attack, owing to the fact that he ate sparingly of the food that gave rise to the poisoning.

A very touching incident occurred in connection with the death of Dr. Wattles, who, fully realizing that he had but a short time to live, desired to see his son married before passing away. The hasty preparation and performance of this ceremony and the receiving of the parental blessing was followed in a short time by the closing of his earthly career.

Dr. Junius H. Wattles, Jr., was associated with his father in the conducting of the college, and will, no doubt, continue the work of his father.

---

Simple Treatment for Parturient Fever. The treatment of Evers, which, to say the least, is unique, consists in insufflating as much air as possible into the quarter of the mammary gland. The principle of the apparatus used consists of a metallic box filled with oakum to purify the air; one end of this box is attached to a rubber tube, with a canula at its end, and the other to the insufflator, which may be a rubber bulb or a small bicycle pump. The canula is first immersed in boiling water.

With this treatment Evers asserted that he has cured eleven out of twelve cases in from one and one-half hours to four and one-quarter hours; he insists that the treatment must not be given too late. The cow should be milked two or three hours after she gets up and commences to eat. Lutz, following Evers, has treated four cases, with success.—*L'Echo Vet.*

